# Prescribing Quality in Medical Specialists in Isfahan, Iran

**Published:** 2013

**Authors:** Gholam-Hossein Sadeghian, Leila Safaeian, Ali-Reza Mahdanian, Solmaz Salami, Javad Kebriaee-Zadeh

**Affiliations:** a *Food and Drug Deputy, Isfahan University of Medical Sciences, Isfahan, Iran. *; b *Department of Pharmacology and Toxicology, School of Pharmacy and Pharmaceutical Sciences, Isfahan University of Medical Sciences, Isfahan, Iran.*

**Keywords:** Drug prescription, Drug utilization, Health care, Quality indicators, Prescribing practice

## Abstract

Rational prescribing increases the quality of health care and patient outcomes. In this study, the quality of drug prescription in specialist physicians in Isfahan province of Iran was assessed for evaluating the rational use of drugs and improving the therapeutic outcomes.

This retrospective survey was conducted on a total of 7999530 prescriptions from all general and specialist physicians. The most frequently prescribed drugs and World Health Organization (WHO) prescribing indicators were evaluated in prescriptions of all medical specialties.

Assessment of prescribing indicators revealed poor-quality prescribing performance by general practitioners including high number of medicines prescribed per clients, wide range of prescribed medicines in each prescription, over-prescribing of antibiotics, corticosteroids and injectable drugs. There were also wide differences in the pattern of drug prescribing depending on the medical specialties. The average number of drugs prescribed per encounter by specialists was less than generalists except for the cardiologists. General practitioners, otorhinolaryngologists and general surgeons prescribed more antibiotics. Orthopedic surgeons and general practitioners were the top prescribers of injectable and corticosteroid drugs. The most frequently prescribed medicine groups varied according to the prescribers’ medical specialty. Analgesics and antipyretics were the most prescribed preparations in general medicine, pediatrics, orthopedics, general surgery and cardiology.

Because of the wide variability in the pattern of drug prescribing depending on the medical specialties, specific performance indicators should be developed for each specific medical specialty for better assessing of prescribing quality in specialist physicians.

## Introduction

Prescription is a critical issue in the rational treatment (1). Rational usage of drugs and proper prescription has significant potential to produce health benefit. Irrational use of medicines has become a global problem in developing and developed countries (2, 3). Over- and under-prescribing, polypharmacy, the use of medicines that are not related to the diagnosis, unnecessary use of expensive medicines and inappropriate use of antibiotics are some examples of irrational prescription (4, 5). 

Inappropriate prescribing may result in treatment failure and reduction of medical care quality, development of drug resistance, increase toxicity risks and loss of faith in medical profession. It also leads to the higher therapeutic costs and wastage of resources (6, 7).

Before any effort for promoting rational prescribing, the quantity of problem should be described. A number of performance indicators have been developed for evaluating the quality of drug use at health facilities by the World Health Organization (WHO). Some prescribing indicators are included: average number and type of prescribed drugs, percentage of antimicrobial and injectable drugs, and percentage of drugs prescribed by generic name and from essential drugs list (8). These quantitative standard indicators have been used in many countries as an assessment method for the evaluation of prescription quality and achieving the regional pattern of drug prescribing in the country. In Iran, national drug policies and regulatory systems including the Rational Use of Drugs (RUD) Committee have been developed to govern the process of prescription.

Although prescribing pattern in general physicians have been reported in various studies, there are only limited data about the medical specialists. The present study aimed to assess the quality of drug prescription in specialist physicians in Isfahan province of Iran for evaluating the rational use of drugs and improving therapeutic outcomes. These prescription data would be helpful in defining problems in drug use patterns and also in identifying the motivating factors and underlying causes including informational, economic, social, cultural and regulatory factors. Moreover, measuring the impact of interventions, making health policies and national drug planning, have been influenced by these drug use patterns (9, 10).

## Experimental

The prescription data from all general and specialist physicians were collected from 20 cities of Isfahan province by RUD Committee in Food and Drug Deputy of Isfahan University of Medical Sciences in 2010. Isfahan province is situated 400 Km of Tehran in center of Iran and there are 4050 general physicians and 1970 specialist physicians in this province. In this retrospective survey, 7999530 prescriptions from Social Security Insurance Organization (an Iranian public insurance organization) issued by general and specialist physicians were investigated.

The following prescribing indicators were determined in prescriptions from general and specialist physicians: average number of drugs per encounter, percentage of prescription with 4 or more preparations, mean cost of prescriptions, percentage of patients receiving antibiotics, percentage of patients receiving injectable drugs, and percentage of patients receiving corticosteroids.

The most frequently prescribed drugs were also evaluated in prescriptions of all physicians. The prescribed drugs were classified according to the AHFS (American Hospital Formulary Service) Pharmacologic-Therapeutic Classification System (2009) (11). The prescriber’s medical specialties was classified as general practice (GP), pediatrics, internal medicine, obstetrics and gynecology, surgery, orthopedics, cardiology, ophthalmology, otorhinolaryngology (ear, nose and throat; ENT), neurology, psychiatry, and others (including infectious diseases, rheumatology, endocrinology, dermatology, gastroenterology, nephrology, pneumology, anesthesiology, *etc*). Medical specialties with lower than 80000 prescriptions per year (about 1% of total prescriptions), were described as “other”.

The prescriptions data were presented as frequency percentage or mean, and analyzed by using professional computer software program (Rx Analyzer). Rx Analyzer or Noskhehpardaz is an Iranian software for gathering and analyzing prescription data. This software has installed in all of pharmacies in the country and has ability to record unlimited number of prescriptions regarding various medicine and also prescriber information. It has capability of analyzing the prescriptions according to the specific prescribing indicators.

*Limitations*

In this study, patients’ characteristics were not recorded. Since the information about the diagnosis for each prescription was not available, the indication for the use of each drug could not be assessed. It is noteworthy that WHO prescribing indicators were not valuable for assessing the quality of medicines prescribed by all specialist physicians including neurology, psychology, endocrinology, etc.

## Results and Discussion

A total of 7999530 prescriptions were studied in this survey. More than half of the prescriptions (4940767 prescriptions; 61.76%) were issued by general practitioners and contained a total of 1332 medicines. The 3058763 prescriptions (38.24%) were from specialist physicians and contained a total of 889 medicines. According to the medical specialties, the most prescriptions (5.2%) were from gynecologists, 4.5% from pediatricians, 3.3% from internal medicine specialists, 2% from orthopedic surgeons, 1.7% from ophthalmologists, 1.5% from psychiatrists, 1.4% from neurologists, 1.3% from ENT specialists, 1.2% from general surgeons, 1.1% from cardiologists and 15% from other specialists.

Table 1 shows the prescribing pattern of general and specialist physicians in Isfahan province of Iran according to the WHO prescribing indicators. The average number of medicines in each prescription varied according to the medical specialty from 2.01 in prescriptions of ophthalmologists to 3.84 in cardiologists. The range of medicines in each prescription was different from 1-10 in prescriptions of pediatricians, orthopedic and general surgeons to 1-18 in general practitioners and other specialists including nephrologists.

**Table 1:** Prescribing pattern of different medical specialist physicians in Isfahan province of Iran according to WHO (World Health Organization) prescribing indicators

	No. of prescriptions	Mean No. of drugs/prescription (range)	Prescription with ≥ 4 preparations (%)	Mean cost (I.R. Rials)	Patients receiving antibiotics (%)	Patients receiving injectables (%)	Patients receiving corticosteroids (%)
GP	4940767	3.34 (1-18)	20	34950	51.2	49.2	26.7
Gynecologists	415119	2.31 (1-12)	3.2	39886	38.5	15.7	3
Pediatricians	359200	2.60 (1-10)	5.2	27714	38.7	12.7	7.2
Internal medicine	262592	2.99 (1-14)	14.7	61083	26.2	18.7	12.5
Orthopedic surgeons	164168	2.51 (1-10)	5.2	30861	9	57	33.7
Ophthalmologists	138718	2.01 (1-15)	0.7	20552	5.7	2.5	3
Psychiatrists	119569	2.65 (1-11)	8.5	70413	2.5	5	1.2
Neurologists	114932	2.76 (1-12)	10	106871	4.7	14.7	7.7
ENT	101938	2.76 (1-12)	7.2	45468	46.5	23.7	18
General surgeons	97201	2.48 (1-10)	5.5	38448	42	20.2	9.5
Cardiologists	90658	3.84 (1-13)	35.5	73430	4.5	5	2.2
Others	1194668	2.84 (1-18)	13.7	100764	34.3	21.7	18.8

GP: general practice; ENT: ear, nose and throat

The percentage of patients which were prescribed 4 or more preparations in each prescription, showed wide variability from 0.7% in ophthalmology to 35.5% in cardiology.

The mean cost of prescription varied widely depending on the prescribers’ specialty and the highest was in neurology (106871 Rials) and the lowest (20552 Rials) in ophthalmology due to the high cost of neurological medicines. It is noteworthy that the total health expenditure is about 5.5% of Iranian GDP (gross domestic product), GDP per capita is 7700 USD and more than 90% of population could access affordable essential medicines in Iran (12).

The general practitioners, ENT specialists and general surgeons prescribed more antibiotics (51.2%, 46.5% and 42%, respectively). Orthopedic surgeons and general practitioners were the top prescribers of injectable drugs (57% and 49.2%, respectively). Orthopedic surgeons and general practitioners were those prescribing more corticosteroid drugs (33.7% and 26.7%, respectively).

These results showed that most drugs were prescribed by general practitioners. There were high number of medicines prescribed per clients and wide range of prescribed medicines in each prescription of general practitioners in Isfahan province. Prescription of antibiotics, corticosteroids and injectable drugs were also frequently and probably inappropriate in general medicine.

Generalists are the first point of contact for patients and treat a wide variety of medical problems and illnesses compared with specialists and these may explain part of the variation in the pattern of prescribing between them (10). Nevertheless, the assessment of prescribing indicators in general medicine revealed that the main areas need further attention to improve the quality of drug prescription. Regarding the mean item of drugs per prescription, our data showed high rate of polypharmacy in general medicine in Iran. There is no universal or standard for this indicator and it is variable from 1.4 to 4.8 in developing countries (13), and 1.3 to 2.2 in developed countries (14). New drug developments, ageing of population and lack of confidence may be contributed in the increasing use of polypharmacy. High level of polypharmacy practice may lead to higher adverse drug reactions, drug-drug interactions and also higher cost (15).

The average number of drugs prescribed per encounter by specialists was less than generalists except for the cardiologists possibly since patients of cardiologists tend to be sicker and have more underlying medical problems (16). The highest percentage of patients which were prescribed 4 or more preparations in each prescription was also in cardiology. Vallano *et al. *have also reported the median number of 5 drugs in prescriptions of cardiologists in Spain (17).

The widest range of prescribed medicines in each prescription was in general medicine and nephrology. Nephrologists also prescribed more medicine as their patients are older and sicker with complicated medical problems.

Our results demonstrated that general practitioners, ENT specialists and general surgeons prescribed more antibiotics. The rate of antibiotic prescription varies from 17.5% to 60% in developing countries (18, 19), and it is much higher than developed countries (20). In another prescribing study in specialists, pediatricians and ophthalmologists preferably prescribed antibiotics (56 and 60.5%, respectively) and the average percentage of antibiotics in the various specialties has been 29% (17). Irrational and overuse of antibiotics is the main driver of resistance. Emergence of antibiotic resistant organisms is a major global public health problem (21). Large volumes of antibiotics are inappropriately prescribed for mild and non-bacterial infections of upper respiratory tract. Irrational prescribing of broad spectrum antibiotics including the third generation cephalosporins also increases the induction of microbial resistance.

Orthopedic surgeons and general practitioners were the top prescribers of injectable drugs, while pneumologists were those prescribing more injectables (10%) in the study of Vallano *et al. *in Spain (16). Our findings showed high percentage of prescriptions involving the injections. Widespread misuse of injectable drugs is a major health problem in many developing countries. From the health point of view, various human and environmental health hazards are associated with unsafe injections. Moreover, unnecessary prescription of an injection causes the charging of a higher fee for service (22, 23).

Our results also revealed that orthopedic surgeons and general practitioners were those prescribing more corticosteroid drugs. In other reports, comparison of general physicians with specialists has also suggested the overuse of long-term oral corticosteroids and underuse of inhaled corticosteroids by GP for acute exacerbations of asthma (24). Unfortunately, over-prescription and overuse of corticosteroids without indication occur during recent years in Iran. Corticosteroids have potential for abuse and misuse both by the physicians and patients (25). Although corticosteroids has been indicated for advanced illness and despite the various adverse effects associated with the prolonged use of these drugs, many patients tend to receive corticosteroids due to the rapid alleviation of their symptoms.

Table 2 shows the most frequently prescribed medicine groups according to the prescribers’ medical specialty. The group of others in medical specialties was excluded from this assess because of non-accumulating patterns of medicine prescribing in various specialties.

**Table 2:** The most frequently prescribed medication classes according to the prescribers’ medical specialty

**Medical Specialty**	**Medication Classes (% of prescriptions)**
**GP**	Analgesics and Antipyretics (10.25), Penicillins (9.25), Antihistamines (9.12), Adrenal Corticosteroids (8.30)
**Gynecologists**	Skin and Mucous Membrane Anti-infectives (11.88), Antianemia Drugs (9.13), Analgesics and Antipyretics (7.47), Replacement Preparations; Calcium salts (7.28)
**Pediatricians**	Analgesics and Antipyretics (11.63), Antihistamines (9.95), Antitussives, Expectorants and Mucolytic Agents (5.90), Penicillins (5.51)
**Internal medicine**	Antiulcer Agents and Acid Suppressants (11.71), Analgesics and Antipyretics (6.93), Antihistamines (5.26), Adrenal Corticosteroids (4.47)
**Orthopedics**	Analgesics and Antipyretics (23.2), Adrenal Corticosteroids (13.75), Vitamins (12.75), Replacement Preparations; Calcium salts (7.11)
**Ophthalmologists**	Ophthalmological Anti-inflammatory Corticosteroids (33.86), Ophthalmological Anti-infectives (22.85), Artificial Tear Solutions and Ocular Lubricants (8.31), Ophthalmological Vasoconstrictors (5.08)
**Psychiatrists**	Antidepressants (24.94), Anticonvulsants (24.45), Antipsychotics (16.89), Anxiolytics, Sedatives and Hypnotics (12.52)
**Neurologists**	Anticonvulsants (22.68), Antidepressants (15.58), Beta-Adrenergic Blocking Agents (8.43), Anxiolytics, Sedatives and Hypnotics (7.69)
**ENT**	Antihistamines (17.24), Penicillins (7.92), ENT Anti-inflammatory Corticosteroids (7.59), Analgesics and Antipyretics (7.45)
**General surgeons**	Analgesics and Antipyretics (19.84), Cephalosporins (8.29), Antiulcer Agents and acid suppressants (5.89), Antiprotozoals (4.31)
**Cardiologists**	Analgesics and Antipyretics (14.25), Beta-Adrenergic Blocking Agents (12.97), Vasodilating Agents (10.99), Antilipemic Agents (9.44)

GP: general practice; ENT: ear, nose and throat

The most prescribed preparations were analgesics and antipyretics in general medicine, pediatrics, orthopedics, general surgery and cardiology, skin and mucous membrane anti-infectives in gynecology, antiulcer agents and acid suppressants in internal medicine, ophthalmological anti-inflammatory corticosteroids in ophthalmology, antidepressants in psychiatry, anticonvulsants in neurology, and antihistamines in ENT.

Acetaminophen was the most prescribed ingredient in general medicine and pediatrics (5.10% and 9.24%, respectively), clotrimazole (8.60%) in gynecology, omeprazole (4.81%) in internal medicine, diclofenac sodium in orthopedic and general surgery (12.89% and 6.79%, respectively), ophthalmic betamethasone (14.9%) in ophthalmology, sodium valproate in psychiatry and neurology (10.80% and 10.34%, respectively), sodium chloride irrigation solution (6.09%) in ENT and acetylsalicylic acid (12.38%) in cardiology.

The pattern of systemic antibacterial prescription according to the prescribers’ medical specialty has been shown in Table 3. Psychiatry, neurology and cardiology were excluded from this assess because of very low prescription of antibacterial agents. The most prescribed antibiotics were penicillins in general medicine, pediatrics, internal medicine and ENT. Cephalosporins were the most prescribed antibiotics in gynecology, ophthalmology, orthopedic and general surgery. It is noteworthy that other anti-infective agents including antiprotozoals (metronidazole, 4.86%) and antifungals (fluconazole, 3.11%) were also frequently prescribed in gynecology.

**Table 3:** The pattern of systemic antibacterial prescription according to the prescribers’ medical specialty as the percentage of antibiotic classes

	**Penicillins**	**Cephalosporins**	**Macrolides**	**Quinolones**	**Sulfonamides**	**Aminoglycosides**	**Others**	**Total**
GP	9.25	4.70	2.03	0.86	0.65	0.18	2.38	20.05
Gynecology	1.71	5.32	1.83	1.37	0.17	0.42	2.35	13.17
Pediatrics	5.51	4.69	1.65	0.19	1.57	0.05	2.28	15.94
Internal -Med	2.22	1.80	1.60	1.46	0.16	0.08	0.76	8.08
Orthopedics	0.66	2.36	0.05	0.58	0.03	0.35	0.12	4.15
Ophthalmology	0.5	0.86	0.12	0.60	0.05	0.08	0.42	2.63
ENT	7.92	5.57	2.67	1.31	0.64	0.28	0.21	18.6
General surgery	3.41	8.29	0.73	2.29	0.22	0.43	0.78	16.15
Other	2.93	2.30	1.58	1.18	0.18	0.13	0.86	9.16

GP: general practice; ENT: ear, nose and throat

Our results highlighted wide differences in the pattern of drug prescribing depending on the medical specialties. Some differences between generalists and specialist physicians have been reported in the quality of care and treatment of specific diseases such as myocardial infarction and depression, in the incidence of drug-drug interactions in their prescriptions and also in the use of diagnostic and therapeutic modalities (9, 26, 27). Specialist physicians may also have prescribing habits that are different from non-specialist physicians.

In this study, WHO recommended prescribing indicators were used for evaluating the quality of drug prescribing. Although these quantitative indicators have been widely used for problem identification and improving the quality of prescribing, they are more appropriate for general aspect of drug utilization. For some specialties such as neurology, psychology and endocrinology, WHO indicators are not helpful for quality evaluation. Specific performance indicators should be developed for each specific medical specialty and standard or rational should be described for them according to the agreed treatment protocol. Recognizing specialist qualifications and their performance are very important to promote public health safety especially because of the role of specialists as the opinion leaders in physicians’ prescription behaviors (28).

During the last decade, various educational, managerial and regulatory strategies have been developed to promote the rational drug use in Iran (29). However most of these activities have been performed concerning general practitioners and more effective intervention approaches are needed targeting all medical specialties for the improvement of prescribing quality in Iran.

## Conclusions

The assessment of prescribing indicators revealed poor-quality prescribing performance by general practitioners in Isfahan province. There were also wide differences in the pattern of drug prescribing depending on the medical specialties. Because of the wide variability in the pattern of drug prescribing depending on the medical specialties, specific performance indicators should be developed for each specific medical specialty for better assessing of prescribing quality in specialist physicians.
